# Optimizing margin status for improving prognosis in patients with oral cavity squamous cell carcinoma: A retrospective study from the two highest-volume Taiwanese hospitals

**DOI:** 10.3389/fonc.2022.1019555

**Published:** 2022-11-14

**Authors:** Chun-Ta Liao, Li-Yu Lee, Shu-Ru Lee, Shu-Hang Ng, Tsang-Wu Liu, Chih-Yen Chien, Jin-Ching Lin, Cheng Ping Wang, Shyuang-Der Terng, Chun-Hung Hua, Tsung-Ming Chen, Wen-Cheng Chen, Yao-Te Tsai, Chung-Jan Kang, Chi-Ying Tsai, Ying-Hsia Chu, Chien-Yu Lin, Kang-Hsing Fan, Hung-Ming Wang, Chia-Hsun Hsieh, Chih-Hua Yeh, Chih-Hung Lin, Chung-Kan Tsao, Tzu-Chen Yen, Nai-Ming Cheng, Tuan-Jen Fang, Shiang-Fu Huang, Li-Ang Lee, Ku-Hao Fang, Yu-Chien Wang, Wan-Ni Lin, Li-Jen Hsin, Yu-Wen Wen

**Affiliations:** ^1^ Department of Otorhinolaryngology, Head and Neck Surgery, Chang Gung Memorial Hospital and Chang Gung University, Taoyuan, Taiwan; ^2^ Department of Pathology, Chang Gung Memorial Hospital and Chang Gung University, Taoyuan, Taiwan; ^3^ Research Service Center for Health Information , Chang Gung University, Taoyuan, Taiwan; ^4^ Department of Diagnostic Radiology, Chang Gung Memorial Hospital and Chang Gung University, Taoyuan, Taiwan; ^5^ National Institute of Cancer Research, National Health Research Institutes, Miaoli, Taiwan; ^6^ Department of Otolaryngology, Chang Gung Memorial Hospital Kaohsiung Medical Center, Chang Gung University College of Medicine, Kaohsiung, Taiwan; ^7^ Department of Radiation Oncology, Changhua Christian Hospital, Changhua, Taiwan; ^8^ Department of Otolaryngology, National Taiwan University Hospital and College of Medicine, Taipei, Taiwan; ^9^ Department of Head and Neck Surgery, Koo Foundation Sun Yat-Sen Cancer Center, Taipei, Taiwan; ^10^ Department of Otorhinolaryngology, China Medical University Hospital, Taichung, Taiwan; ^11^ Department of Otolaryngology, Shuang Ho Hospital, Taipei Medical University, New Taipei City, Taiwan; ^12^ Department of Radiation Oncology, Chang Gung Memorial Hospital and Chang Gung University, Taoyuan, Taiwan; ^13^ Department of Otorhinolaryngology-Head and Neck Surgery, Chang Gung Memorial Hospital, Chiayi, Taiwan; ^14^ Department of Oral and Maxillofacial Surgery, Chang Gung Memorial Hospital, Chang Gung University, Taoyuan, Taiwan; ^15^ Department of Medical Oncology, Chang Gung Memorial Hospital and Chang Gung University, Taoyuan, Taiwan; ^16^ Department of Plastic and Reconstructive Surgery, Chang Gung Memorial Hospital and Chang Gung University, ROC, Taoyuan, Taiwan; ^17^ Department of Nuclear Medicine and Molecular Imaging Center, Chang Gung Memorial Hospital and Chang Gung University, Taoyuan, Taiwan; ^18^ Clinical Informatics and Medical Statistics Research Center, Chang Gung University, Taoyuan, Taiwan; ^19^ Division of Thoracic Surgery, Chang Gung Memorial Hospital, Taoyuan, Taiwan

**Keywords:** oral cavity squamous cell carcinoma, volume-outcome, hospital volumes, cancer registry, propensity score matching, survival outcomes

## Abstract

**Background:**

In the treatment of oral cavity squamous cell carcinoma (OCSCC), surgical quality measures which are expected to affect outcomes, including the achievement of a clear margin, are surgeon-dependent but might not be invariably associated with hospital volume. Our objective was to explore surgical margin variations and survival differences of OCSCC between two highest-volume hospitals in Taiwan.

**Materials and methods:**

A total of 2009 and 1019 patients with OCSCC who were treated at the two highest-volume Taiwanese hospitals (termed Hospital 1 and Hospital 2, respectively) were included. We examined how a pathological margin <5 mm impacted patient outcomes before and after propensity score (PS) matching.

**Results:**

The prevalence of margins <5 mm was markedly lower in Hospital 1 than in Hospital 2 (34.5%/65.2%, *p*<0.0001). Compared with Hospital 2, tumor severity was higher in Hospital 1. On univariable analysis, being treated in Hospital 2 (versus Hospital 1; hazard ratio [HR] for 5-year disease-specific survival [DSS] = 1.34, *p*=0.0002; HR for 5-year overall survival [OS] = 1.17, *p*=0.0271) and margins <5 mm (versus ≥5 mm; HR for 5-year DSS = 1.63, *p*<0.0001; HR for 5-year OS = 1.48, *p*<0.0001) were identified as adverse factors. The associations of treatment in Hospital 2 and margins <5 mm with less favorable outcomes remained significant after adjustment for potential confounders in multivariable analyses, as well as in the PS-matched cohort. The 5-year survival differences between patients operated in Hospital 1 and Hospital 2 were even more pronounced in the PS-matched cohort (before PS matching: DSS, 79%/74%, *p*=0.0002; OS, 71%/68%, *p*=0.0269; after PS matching: DSS, 84%/72%, *p*<0.0001; OS, 75%/66%, *p*<0.0001). In the entire cohort, the rate of adjuvant therapy was found to be lower in patients with margins ≥5 mm than in those with margins <5 mm (42.7%*/*57.0%, *p*<0.0001).

**Conclusions:**

Within the two highest-volume hospitals in Taiwan, patients with OCSCC with a clear margin status (≥5 mm) achieved more favorable outcomes. These results have clinical implications and show how initiatives aimed at improving the margin quality can translate in better outcomes. A clear margin status can reduce the need for adjuvant therapy, ultimately improving quality of life.

## Introduction

When a hospital volume-outcome relationship exists, centralization of surgical activities is recommended (1). Although a statistically significant relationship has been reported between hospital volume and survival in patients with head and neck cancer (2, 3), a comprehensive examination of whether the same association exists in oral cavity squamous cell carcinoma (OCSCC) is still lacking (3, 4).

The high burden of oral malignancies in Asian countries is well established, and the Taiwan Health Promotion Administration (THPA) has formulated quality standards for hospitals performing OCSCC surgery (5). According to the National Comprehensive Cancer Network (NCCN) guidelines released in 2020, the presence of margins <5 mm (positive or close margins) is considered an adverse prognostic variable in patients with OCSCC (6). Recent (2020) quality criteria mandated by the THPA include the achievement of a clear margin status (≥5 mm) and an adequate neck node yield (≥15 lymph nodes). In the treatment of OCSCC, these quality measures, which are expected to affect clinical outcomes, are surgeon-dependent but might not be invariably associated with hospital volume. Of note, a clear margin can sometimes reduce the need for postoperative adjuvant therapy, ultimately improving quality of life.

The purpose of the current study was to explore margin status variations and differences in survival between the two highest-volume Taiwanese hospitals that perform surgical excision of OCSCC. To this aim, we examined how margins <5 mm impacted patient outcomes. We hypothesized that there would be significant variation between hospitals and that surgical margins – as a quality standard – would be associated with clinical outcomes after adjustment for clinicopathological risk factors (RFs) and treatment modalities (7).

## Materials and methods

### Data sources

The present retrospective analysis was based on data obtained from the Taiwanese Cancer Registry Database (TCRD) “long-form”, which is a nationwide dataset that prospectively includes information about cancer stage, disease relapses, and treatment modalities. Collection of pathological data on margin status, tumor depth of invasion (DOI), and extra-nodal extension (ENE) began in 2011. The aim of the TCRD is to include clinical and pathological information from patients with malignancies admitted to major Taiwanese hospitals; the vast majority (>99%) of patients with OCSCC in the country are included in this registry. Follow-up data collected from the Taiwanese National Health Insurance Research Dataset (TNHIRD) were used to determine survival outcomes. This study follows the reporting recommendations for tumor marker prognostic studies (REMARK) (8, 9). The study protocol was reviewed and approved by the Institutional Review Board of the Chung Gung Memorial Hospital (reference number: 201801398B0A3) and received a waiver of patient consent.

### Treatment protocol and follow-up protocol

As part of its continued effort to improve the quality of cancer care, the THPA has taken initiative to promote multidisciplinary team care (MDTC) and multidisciplinary case management as of April 2003. Because outcomes in patients with OCSCC are largely dependent on the type of surgical approach and the use of adjuvant therapy, a comprehensive strategy for decision-making, therapy, clinical management, and follow-up is mandatory in areas where betel quid chewing is endemic. Starting from these premises, all of the Taiwanese hospital specialized in treating OCSCC began implementing an MDTC approach as of January 2004. In general, each hospital’s treatment and follow-up protocols were in accordance with the NCCN treatment guidelines (6).

### Data collection

In the TCRD, patient staging was originally performed using the AJCC Staging Manual, seventh edition (2010). Disease stages were subsequently updated according to the AJCC Staging Manual, eight edition (2018), by integrating information on DOI and ENE (10). The study data were downloaded from the TCRD (2018 release) and TNHIRD (2019 release) and last analyzed in October 2022. Information on OCSCC-related morbidity and mortality obtained from the TNHIRD was used to calculate disease-specific survival (DSS) and overall survival (OS). The TCRD follows the guidance for registries outlined in the Standards for Oncology Registry Entry (STORE) manual (11). According to the STORE guidelines, data concerning local, regional, and distant recurrences are recorded concomitantly for one location rather than by taking into account the three locations separately; in addition, only the first recurrence was recorded. Information from each hospital was transmitted to the TCRD during the first and fifth years of follow-up. In contrast, TNHIRD data are updated on an annual basis. In light of this methodology, survival data can be considered entirely reliable for the calculation of DSS and OS, whereas this might not be the case for disease-free survival (including calculation of local control, neck control, and distant metastases).

### Patient selection

**Figure 1:**
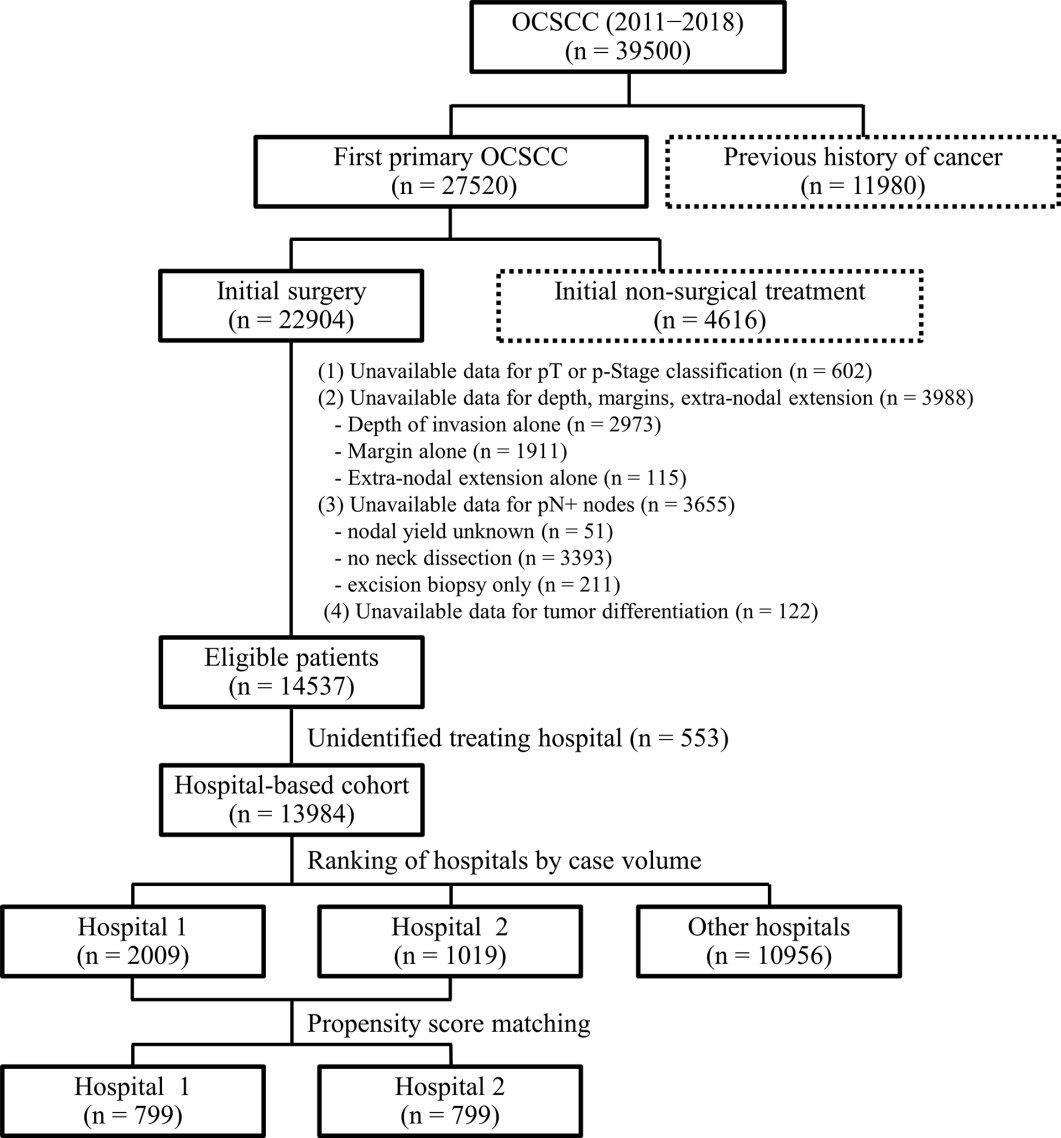
Flow of patients through the study.

Patients included in the TCRD over an 8-year period (2011−2018) were eligible if they had a documented diagnosis of first primary lip (ICDO-3 codes: C00.0−C00.9) and oral cavity (ICDO-3 codes: C02.0−C06.9) squamous cell carcinoma. The follow-up concluded in December 2019. Patients with a previous history of cancer (n = 11980), initially treated with non-surgical modalities (n = 4616), and with an unknown pathological stage (n = 602) were excluded, as were those with unavailable data concerning tumor depth, surgical margins, and ENE (n = 3988), pathological nodal metastases (n = 3655), tumor differentiation (n = 122), and treating hospital (n = 553). Finally, a total of 13984 patients admitted to 75 different hospitals were identified. The study flow chart is shown in **Figure 1**.

### Hospital-based analysis of surgical margin status

**Figure 2:**
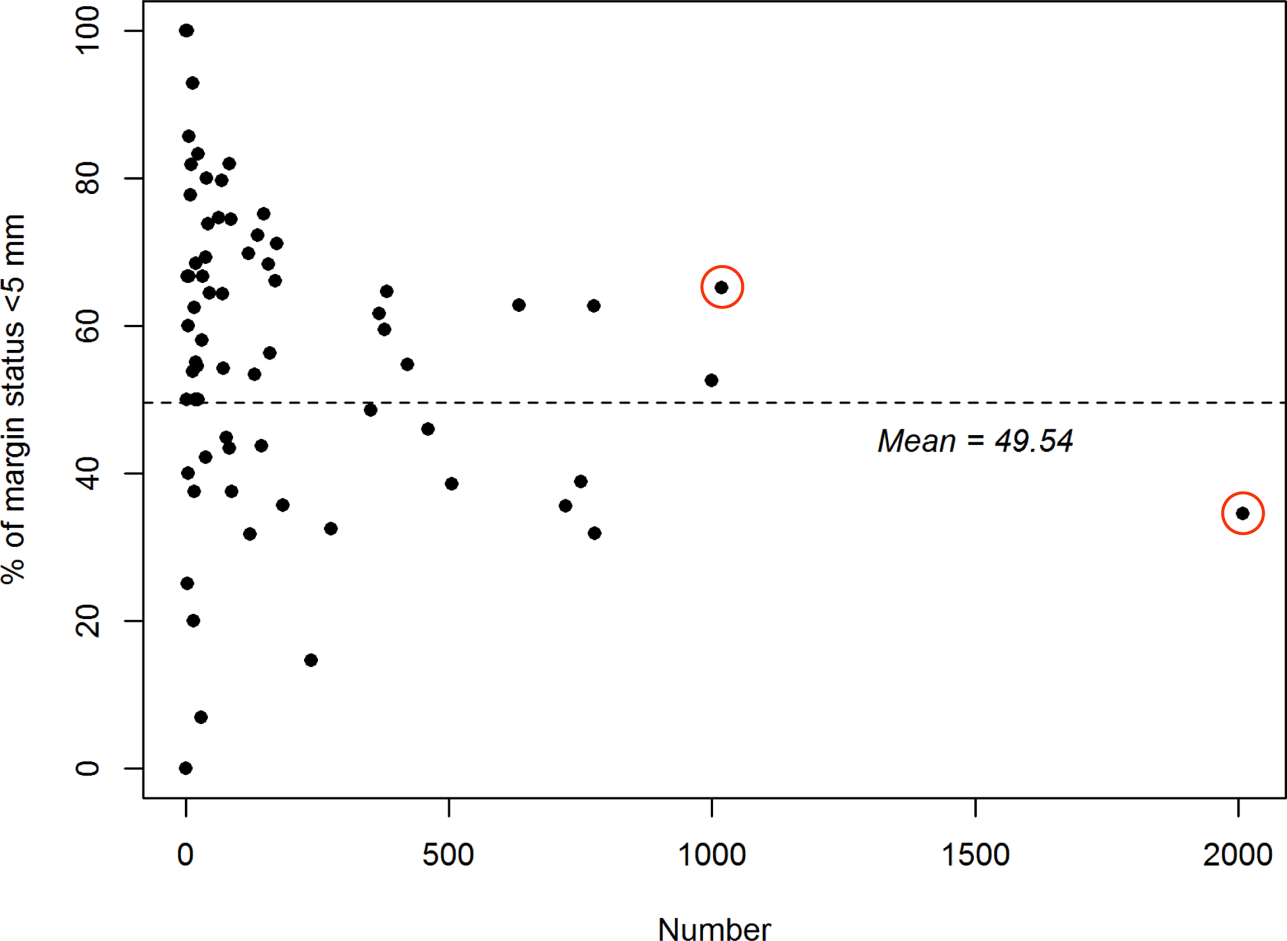
Relationship between hospital volume (plotted on the x-axis; scale: number of treated patients) and the proportion of patients with margins <5 mm (plotted on the y-axis) in 75 Taiwanese hospitals specializing in the treatment of oral cavity squamous cell carcinoma. The red circles denote the two hospitals included in the study.

The frequencies of patients with surgical margins <5 mm according to the number of treated cases in each hospital is shown in **Figure 2**. On average, 49.5% of the study participants had margins of less than 5 mm. A total of 2009 and 1019 patients were treated at the two highest volume hospitals (termed Hospital 1 and Hospital 2, respectively). Notably, the prevalence of margins <5 mm was markedly lower in former than in the latter (34.5% and 65.2%, respectively), prompting the need for a comprehensive assessment of the prognostic impact of surgical margins in OCSCC.

Therefore, data from these two hospitals were subjected to further analysis.

### Statistical analysis

The outcome measures for this study were the 5-year DSS and OS rates. The follow-up duration was defined as the time between the date of surgery to the date of death or date of study termination (December 2019). Except for surgical margins, propensity score (PS) matching with logistic regression was performed to balance the baseline covariates between Hospital 1 and Hospital 2. In PS matching, the balance in measured covariates was evaluated by calculating the standardized mean differences (SMD) for the matched cohort (12). An SMD of less than 10% suggested a good balance in baseline variables. The clinical outcomes of patients enrolled from Hospital 1 and Hospital 2 were analyzed before and after PS matching. Survival probabilities were graphically represented with Kaplan-Meier curves (log-rank test). The associations between the study variables and survival outcomes were modeled by multivariable Cox proportional hazards regression analysis after adjusting for all covariates entered in univariable analysis. In this model, a multivariable stepwise selection procedure was applied and the results were expressed as hazard ratios (HRs) and 95% confidence intervals (CIs). All analyses were performed using R, version 4.0.2 (R Foundation for Statistical Computing, Vienna, Austria) and SAS, version 9.4 (SAS Institute Inc., Cary, NC, USA). Two-sided hypotheses were adopted for statistical testing.

## Results

### Patient characteristics

**Table 1** lists the general characteristics of the study patients treated in the two highest-volume hospitals in Taiwan. The number of patients treated in Hospital 1 was nearly two-fold higher than those treated in Hospital 2. In addition, the number of patients treated in each hospital remained relatively stable throughout the study period for both Hospital 1 (number of treated patients: year 2011, n = 254; year 2012, n = 287; year 2013, n = 218; year 2014, n = 246; year 2015, n = 239; year 2016, n = 230; year 2017, n = 241; and year 2018, n = 294) and Hospital 2 (number of treated patients: year 2011, n = 128; year 2012, n = 130; year 2013, n = 132; year 2014, n = 128; year 2015, n = 128; year 2016, n = 123; year 2017, n = 130; and year 2018, n = 120, *p* = 0.281). Before PS matching, several RFs – including margin status, tumor subsite, sex, age, pathological T status, pathological N status, pathological stage, tumor differentiation, DOI, ENE, neck nodal yield, and treatment modality – differed significantly between the two hospitals. Compared with Hospital 2, tumor severity was higher in Hospital 1. With the exception of surgical margins, all covariates had a SMD <10% after PS matching (n = 799 in each group), demonstrating adequate balance between the two hospitals.

**Table 1:** General characteristics of patients with oral cavity squamous cell carcinoma enrolled from Hospital 1 and Hospital 2 before (n = 3028) and after (n = 1598) propensity score matching.

	Before PS matching				After PS matching		
Characteristic (n, %; before PS matching)	Hospital 1 (n = 2009)n (%)	Hospital 2 (n = 1019)n (%)	SMD (%)	*p ^a^*	Hospital 1 (n = 799)n (%)	Hospital 2 (n = 799)n (%)	SMD (%)
Margin status (mm)				<0.0001			
<5 (1357, 44.8)	693 (34.5)	664 (65.2)	-64.44		276 (34.5)	528 (66.1)	-66.47
≥5 (1671, 55.2)	1316 (65.5)	355 (34.8)	64.44		523 (65.5)	271 (33.9)	66.47
Tumor subsite				<0.0001			
Lip (99, 3.3)	74 (3.7)	25 (2.5)	7.14		20 (2.5)	21 (2.6)	-0.79
Tongue (1272, 42.0)	775 (38.6)	497 (48.8)	-20.67		368 (46.1)	371 (46.4)	-0.75
Gum (386, 12.7)	267 (13.3)	119 (11.7)	4.88		87 (10.9)	92 (11.5)	-1.98
Mouth floor (129, 4.3)	91 (4.5)	38 (3.7)	4.02		36 (4.5)	35 (4.4)	0.61
Hard palate (48, 1.6)	24 (1.2)	24 (2.4)	-8.8		16 (2.0)	15 (1.9)	0.97
Buccal (888, 29.3)	625 (31.1)	263 (25.8)	11.77		231 (28.9)	218 (27.3)	3.62
Retromolar (140, 4.6)	104 (5.2)	36 (3.5)	8.06		27 (3.4)	31 (3.9)	-2.68
Other sites (66, 2.2)	49 (2.4)	17 (1.6)	5.44		14 (1.7)	16 (2.0)	-1.84
Sex				<0.0001			
Men (2703, 89.3)	1843 (91.7)	860 (84.4)	22.79		702 (87.9)	705 (88.2)	-1.16
Women (325, 10.7)	166 (8.3)	159 (15.6)	-22.79		97 (12.1)	94 (11.8)	1.16
Age (years)				0.0003			
<65 (2511, 82.9)	1701 (84.7)	810 (79.5)	13.54		646 (80.9)	662 (82.9)	-5.2
≥65 (517, 17.1)	308 (15.3)	209 (20.5)	-13.54		153 (19.1)	137 (17.1)	5.2
Pathologic T status				<0.0001			
T1 (587, 19.4)	328 (16.3)	259 (25.4)	-22.51		189 (23.7)	180 (22.5)	2.67
T2 (962, 31.8)	620 (30.9)	342 (33.6)	-5.78		252 (31.5)	262 (32.8)	-2.68
T3 (468, 15.5)	313 (15.6)	155 (15.2)	1.02		137 (17.1)	129 (16.1)	2.69
T4 (1011, 33.4)	748 (37.2)	263 (25.8)	24.77		221 (27.7)	228 (28.6)	-1.95
Pathologic N status				0.0009			
pN0 (1988, 65.7)	1301 (64.8)	687 (67.4)	-5.62		546 (68.3)	535 (67.0)	2.94
pN1 (309, 10.2)	194 (9.7)	115 (11.3)	-5.32		93 (11.6)	88 (11.0)	1.97
pN2 (272, 9.0)	173 (8.6)	99 (9.7)	-3.83		64 (8.0)	75 (9.4)	-4.89
pN3 (459, 15.2)	341 (16.9)	118 (11.6)	15.46		96 (12.1)	101 (12.6)	-1.9
Pathologic stage				<0.0001			
I (518, 17.1)	291 (14.5)	227 (22.3)	-20.22		166 (20.8)	159 (19.9)	2.18
II (705, 23.3)	464 (23.1)	241 (23.7)	-1.31		179 (22.4)	187 (23.4)	-2.38
III (465, 15.4)	302 (15.0)	163 (16.0)	-2.66		148 (18.5)	130 (16.3)	5.95
IV (1340, 44.3)	952 (47.4)	388 (38.0)	18.9		306 (38.3)	323 (40.4)	-4.36
Tumor differentiation				<0.0001			
Well (812, 26.8)	375 (18.7)	437 (42.9)	-54.38		286 (35.8)	296 (37.0)	-2.6
Moderately (1902, 62.8)	1359 (67.6)	543 (53.3)	29.69		473 (59.2)	465 (58.2)	2.03
Poorly (314, 10.4)	275 (13.7)	39 (3.8)	35.43		40 (5.0)	38 (4.8)	1.16
Depth of invasion (mm)				<0.0001			
<10 (1554, 51.3)	971 (48.3)	583 (57.2)	-17.86		444 (55.6)	436 (54.6)	2.01
≥10 (1474, 48.7)	1038 (51.7)	436 (42.8)	17.86		355 (44.4)	363 (45.4)	-2.01
Extra-nodal extension				0.0006			
No (2564, 84.7)	1669 (83.1)	895 (87.8)	-13.52		706 (88.4)	700 (87.6)	2.31
Yes (464, 15.3)	340 (16.9)	124 (12.2)	13.52		93 (11.6)	99 (12.4)	-2.31
Neck nodal yield				<0.0001			
<15 nodes (228, 7.5)	35 (1.7)	193 (18.9)	-58.88		35 (4.4)	30 (3.8)	3.17
≥15 nodes (2800, 92.5)	1974 (98.3)	826 (81.1)	58.88		764 (95.6)	769 (96.2)	-3.17
Treatment modality				0.0075			
S alone (1540, 50.9)	987 (49.1)	553 (54.3)	-10.3		466 (58.3)	444 (55.6)	5.56
S plus adjuvant therapy^b^ (1488, 49.1)	1022 (50.9)	466 (45.7)	10.3		333 (41.7)	355 (44.4)	-5.56

PS, propensity score; S, surgery; CT, chemotherapy; RT, radiotherapy; SMD, standardized mean difference.

^a^Chi-square test; ^b^S plus CT + S plus RT+ S plus CT and RT.

**Figure 3:**
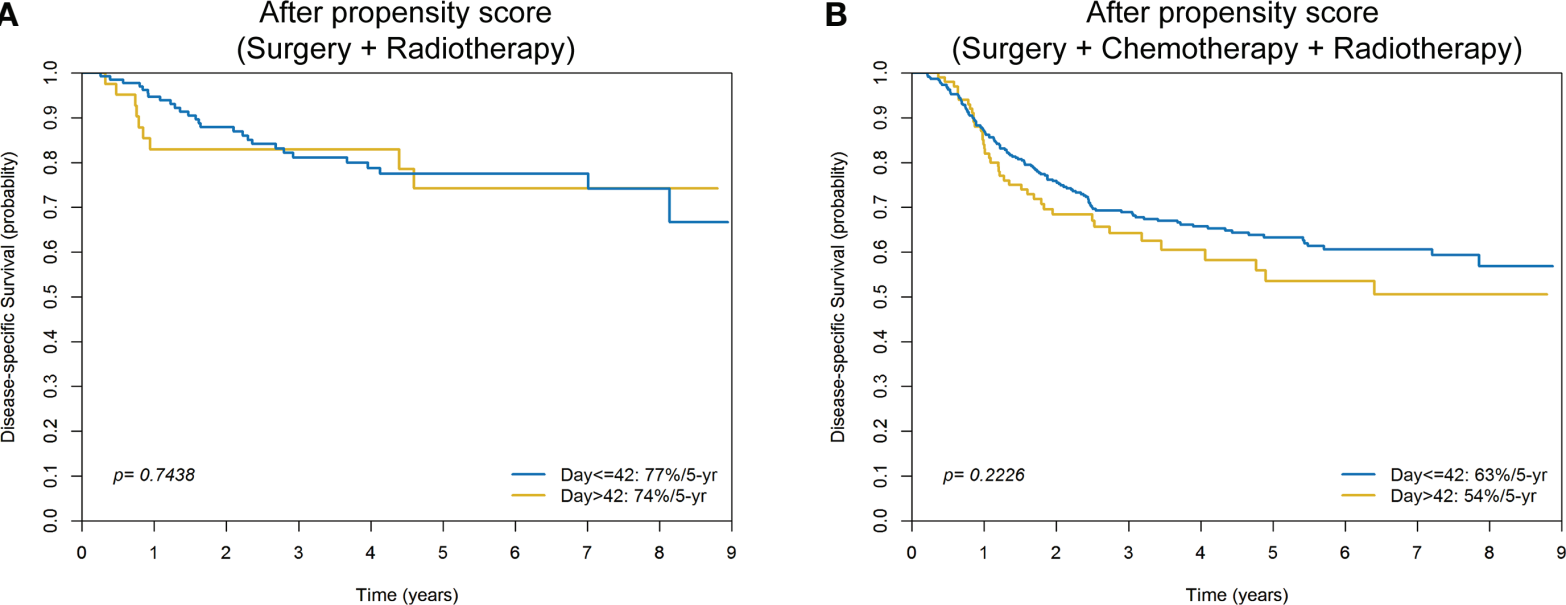
Kaplan-Meier plots comparing 5-year disease-specific survival of surgery-to-radiotherapy intervals ≤42 days versus >42 days for patients with oral cavity squamous cell carcinoma treated with surgery plus radiotherapy **(A)** or surgery plus chemotherapy plus radiotherapy **(B)** after propensity score matching.

**Table 2** summarizes the radiotherapy (RT) characteristics in the adjuvant group of patients. There were significant differences between the two hospitals in the use of RT techniques, radiation doses, and surgery-to-radiation intervals. Compared with Hospital 1, the surgery-to-RT interval was longer in Hospital 2. However, in the propensity score group, there were no survival differences between surgery-to-RT intervals ≤42 days and >42 days, either in the adjuvant RT subgroup (n = 172, 5-year DSS, *p* = 0.7438, **Figure 3A**) or in the adjuvant chemotherapy plus RT subgroup (n = 476, 5-year DSS, *p* = 0.2226, **Figure 3B**).

**Table 2:** General characteristics of patients with oral cavity squamous cell carcinoma receiving adjuvant radiotherapy therapy enrolled from Hospital 1 and Hospital 2 before (n = 1406) and after (n = 648) propensity score matching.

	Before PS matching			After PS matching		
Characteristic (n, %; before PS matching)	Hospital 1 n (%)	Hospital 2 n (%)	*p*	Hospital 1 n (%)	Hospital 2 n (%)	*p*
RT Technique in S+RT Subgroup (n = 390)			0.3963^a^			0.4333^a^
Conformal (10)	10 (3.0)	0 (0.0)		3 (2.3)	0 (0.0)	
IMRT (100)	84 (25.3)	16 (27.6)		33 (25.6)	14 (32.6)	
VMAT (280)	238 (71.7)	42 (72.4)		93 (72.1)	29 (67.4)	
RT dose (cGy) in S+RT subgroup (n = 390)						
Range (200-8200)	200-8200	800-7100		200-8200	800-7100	
Median (6000)	6000	6600	<0.0001^b^	6000	6600	0.0007^b^
Mean (6037.5)	5987.0	6326.6		5905.1	6245.1	
S-to-RT interval in S+RT subgroup (n = 390)						
Range (20-90 days)	20-78	26-90		20-78	26-90	
Median 40 days	40	41	0.0195^b^	40	43	0.0017^b^
Mean 39.4 days	38.8	42.7		38.6	44.9	
RT Technique in S+CT+RT subgroup (n = 1016)			<0.0001^a^			0.0091^a^
Conformal (7)	8 (1.2)	0 (0.0)		2 (1.0)	0 (0.0)	
IMRT (244)	139 (20.3)	105 (31.7)		37 (18.3)	79 (28.8)	
VMAT (764)	538 (78.5)	226 (68.3)		163 (80.7)	195 (71.2)	
RT dose (cGy) in S+CT+RT subgroup (n = 1016)						
Range (700-8800)	700-8800	1200-7060		1200-8800	1200-7060	
Median (6600)	6600	6600	0.0007^b^	6600	6600	0.1569^b^
Mean (6480.7)	6519.0	6401.6		6471.1	6386.9	
S-to-RT interval in S+CT+RT subgroup (n = 1016)						
Range (13-210 days)	13-168	19-210		13-168	19-210	
Median 39 days	39	40	<0.0001^b^	39	40	<0.0001^b^
Mean 39.0 days	37.5	42.1		38.0	42.8	

PS, propensity score; S, surgery; CT, chemotherapy; RT, radiotherapy; IMRT, intensity-modulated radiation therapy; VMAT, **volumetric modulated arc therapy.**

^a^Chi-square test; ^b^Mann-Whitney U Test (skewed data).

### Univariable and multivariable cox regression analysis

Unadjusted univariable analyses identified several RFs as being associated with less favorable 5-year DSS and OS (**Table 3**). Of note, the results revealed that being treated in Hospital 2 (versus Hospital 1; unadjusted HR for 5-year DSS = 1.34, *p* = 0.0002; unadjusted HR for 5-year OS = 1.17, *p* = 0.0271) and margins <5 mm (versus margins ≥5 mm; unadjusted HR for 5-year DSS = 1.63, *p <*0.0001; unadjusted HR for 5-year OS = 1.48, *p <*0.0001) were adverse prognostic factors. The associations of treatment in Hospital 2 and margins <5 mm with adverse outcomes remained significant after adjusting for potential confounders in multivariable analysis (**Table 3**).

**Table 3:** Univariable and multivariable analyses of risk factors for 5-year disease-specific and overall survival rates in patients with oral cavity squamous cell carcinoma (n = 3028) enrolled from Hospital 1 and Hospital 2.

Risk factor	Disease-specific survival								Overall survival							
		Univariable analysis				Stepwise multivariable analysis				Univariable analysis				Stepwise multivariable analysis			
		HR (95% CI)		*p*		HR (95% CI)		*p*		HR (95% CI)		*p*		HR (95% CI)		*p*	
Hospital																
Hospital 1	1				1				1				1			
Hospital 2	1.34 (1.15-1.57)		0.0002		1.94 (1.62-2.33)		<0.0001		1.17 (1.02-1.34)		0.0271		1.54 (1.32-1.81)		<0.0001	
Margin status (mm)																
<5	1.63 (1.40-1.90)		<0.0001		1.18 (1.001-1.39)		0.0489		1.48 (1.29-1.68)		<0.0001		1.16 (1.01-1.34)		0.0335	
≥5	1				1				1				1		
Tumor subsite																
Lip	1				–				1				–		
Tongue	1.12 (0.71-1.76)		0.6259		–		ns		1.01 (0.69-1.47)		0.9811		–		ns	
Gum	1.21 (0.75-1.97)		0.4368		–		ns		1.24 (0.83-1.86)		0.2905		–		ns	
Mouth floor	0.88 (0.48-1.60)		0.6773		–		ns		0.96 (0.59-1.56)		0.8567		–		ns	
Hard palate	1.31 (0.65-2.64)		0.4436		–		ns		1.45 (0.83-2.55)		0.1922		–		ns	
Buccal	1.02 (0.64-1.62)		0.9299		–		ns		0.92 (0.63-1.36)		0.6876		–		ns	
Retromolar	1.13 (0.64-1.98)		0.6755		–		ns		1.06 (0.66-1.69)		0.8151		–		ns	
Other sites	1.87 (1.03-3.38)		0.0394		–		ns		1.68 (1.01-2.79)		0.0441		–		ns	
Sex																
Men	1.03 (0.80-1.33)		0.8202		–		ns		1.11 (0.89-1.39)		0.3527		–		ns	
Women	1				–				1				–		
Age (years)																
<65	1				1				1				1		
≥65	1.20 (0.98-1.46)		0.0776		1.37 (1.12-1.68)		0.0024		1.40 (1.19-1.65)		<0.0001		1.62 (1.37-1.92)		<0.0001	
Pathologic T status																
T1	1				1				1				1		
T2	1.37 (1.02-1.85)		0.0364		1.12 (0.82-1.52)		0.4744		1.42 (1.12-1.81)		0.0043		1.27 (0.992-1.62)		0.0580	
T3	2.66 (1.96-3.60)		<0.0001		1.39 (0.94-2.07)		0.1028		2.39 (1.86-3.08)		<0.0001		1.81 (1.39-2.35)		<0.0001	
T4	3.72 (2.84-4.87)		<0.0001		1.93 (1.32-2.81)		0.0007		3.43 (2.75-4.29)		<0.0001		2.58 (2.03-3.26)		<0.0001	
Pathologic N status																
pN0	1				1				1				1		
pN1	2.06 (1.60-2.65)		<0.0001		1.59 (1.22-2.06)		0.0005		1.97 (1.59-2.43)		<0.0001		1.58 (1.27-1.97)		<0.0001	
pN2	2.67 (2.08-3.42)		<0.0001		1.89 (1.46-2.44)		<0.0001		2.39 (1.93-2.95)		<0.0001		1.82 (1.46-2.27)		<0.0001	
pN3	5.77 (4.83-6.90)		<0.0001		3.64 (2.98-4.45)		<0.0001		4.95 (4.24-5.78)		<0.0001		3.36 (2.83-3.99)		<0.0001	
Pathologic stage																
I	1				–				1				–		
II	1.07 (0.74-1.56)		0.7051		–		ns		1.23 (0.91-1.65)		0.1728		–		ns	
III	2.22 (1.56-3.16)		<0.0001		–		ns		2.20 (1.64-2.93)		<0.0001		–		ns	
IV	4.59 (3.40-6.21)		<0.0001		–		ns		4.22 (3.30-5.41)		<0.0001		–		ns	
Tumor differentiation																
Well differentiated	1				1				1				1		
Moderately differentiated	1.89 (1.53-2.33)		<0.0001		1.63 (1.30-2.04)		<0.0001		1.67 (1.41-1.98)		<0.0001		1.45 (1.21-1.75)		<0.0001	
Poorly differentiated	3.31 (2.54-4.31)		<0.0001		2.37 (1.77-3.18)		<0.0001		2.77 (2.22-3.47)		<0.0001		2.00 (1.56-2.56)		<0.0001	
Depth of invasion (mm)																
<10	1				1				1				–		
≥10	2.78 (2.35-3.29)		<0.0001		1.42 (1.08-1.86)		0.0120		2.41 (2.10-2.76)		<0.0001		–		ns	
Extra-nodal spread																
No	1				–				1				–		
Yes	3.84 (3.26-4.51)		<0.0001		–		ns		3.40 (2.95-3.92)		<0.0001		–		ns	
Neck nodal yield																
<15 nodes	1				–				1				–		
≥15 nodes	1.25 (0.91-1.70)		0.1687		–		ns		1.09 (0.85-1.41)		0.5062		–		ns	
Treatment modality																
S alone	1				–				1				–		
S plus adjuvant therapy^a^	2.40 (2.04-2.83)		<0.0001		–		ns		2.08 (1.82-2.39)		<0.0001		–		ns	

S, surgery; CT, chemotherapy; RT, radiotherapy; ns, not significant; HR, hazard ratio; CI, confidence interval; ns, not significant.

^a^S plus CT + S plus RT+ S plus CT and RT.

### Outcome analysis

**Figure 4:**
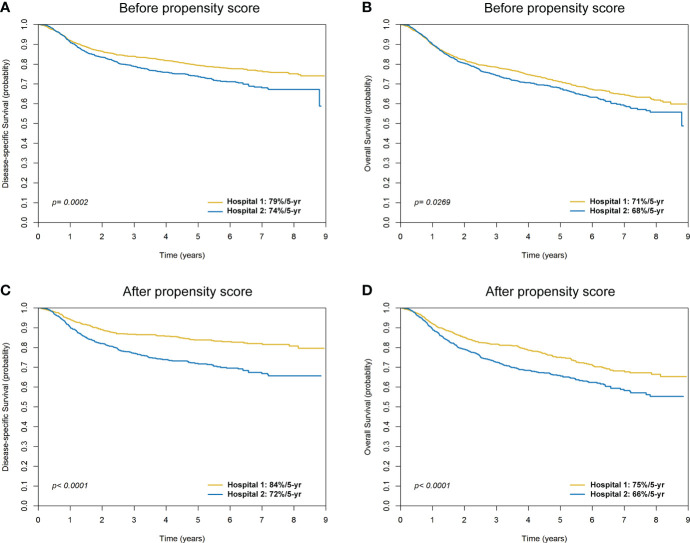
Kaplan-Meier plots comparing 5-year disease-specific survival and overall survival for patients with oral cavity squamous cell carcinoma in Hospital 1 and Hospital 2 before **(A, B)** and after **(C, D)** propensity score matching.

Before PS matching, the 5-year DSS and OS rates in patients treated in Hospital 1 were higher than those observed for patients treated in Hospital 2 (79%/74%, *p* = 0.0002; and 71%/68%, *p* = 0.0269; respectively; **Figures 4A, B**). In the PS-matched cohort, the survival differences between patients operated by Hospital 1 and Hospital 2 were even more pronounced: 5-year DSS (84%/72%, HR = 1.89, *p <*0.0001) and OS (75%/66%, HR = 1.45, *p <*0.0001) (**Figures 4C, D**; **Table 4**).

**Table 4:** Five-year disease-specific and overall survival rates in patients with oral cavity squamous cell carcinoma enrolled from Hospital 1 and Hospital 2 after propensity score matching (n = 1598).

	Disease-specific survival		Overall survival	
	HR (95% CI)	*p*	HR (95% CI)	*p*
Hospital 1	1		1	
Hospital 2	1.89 (1.51-2.36)	<0.0001	1.45 (1.21-1.75)	<0.0001

HR, hazard ratio; CI, confidence interval.

### Impact of margin status on five-year outcomes in the internal grouping (pathological stage and treatment modality) of patients enrolled from the two hospitals

The 5-year DSS rates of patients with margins ≥5 mm versus <5* mm* were 82%/74% in Hospital 1 (*p <*0.0001) and 80%/70% in Hospital 2 (*p* = 0.0006). The 5-year OS rates of patients with margins ≥5 mm versus <5* mm* were 75%/64% in Hospital 1 (*p <*0.0001) and 75%/64% in Hospital 2 (*p* = 0.0010; **Table 5**). Pathological stage (pStage I-II versus pStage III-IV) and treatment modalities (surgery alone versus surgery plus adjuvant therapy) were stratified for further analyses. Marginal status (≥5 mm versus <5* mm*) had a more significant impact on 5-year survival rates in patients with pStage III-IV disease (compared with pStage I-II) and in those treated with surgery plus adjuvant therapy (compared with surgery alone) in both hospitals (**Table 5**).

**Table 5:** Impact of surgical margin status on five-year disease-specific and overall survival rates in the internal grouping (pathological stage and treatment modality) of Hospital 1 and Hospital 2.

Characteristic (n, %)	Five-year disease-specific survival(margin ≥5 mm vs. <5 mm)	*p*	Five-year overall survival(margin ≥5 mm vs. <5 mm)	*p*
**Hospital 1** (n = 2009)	82% / 74%	<0.0001	75% / 64%	<0.0001
Pathological stage				
I-II (755, 37.6)	92% / 92%	0.9690	87% / 86%	0.8202
III-IV (1254, 62.4)	74% / 67%	0.0030	66% / 55%	0.0013
Treatment modality				
Surgery (987, 49.1)	88% / 86%	0.3469	81% / 77%	0.2758
Surgery plus adjuvant therapy (1022, 50.9)	74% / 68%	0.0278	66% / 58%	0.0229
**Hospital 2** (n = 1019)	80% / 70%	0.0006	75% / 64%	0.0010
Pathological stage				
I-II (468, 45.9)	90% / 87%	0.2411	88% / 82%	0.1856
III-IV (551, 54.1)	68% / 58%	0.0161	60% / 50%	0.0327
Treatment modality				
Surgery (553, 54.3)	88% / 79%	0.0409	83% / 73%	0.0486
Surgery plus adjuvant therapy (466, 45.7)	70% / 61%	0.0089	65% / 54%	0.0091

Among patients who were treated with surgery alone, the margin status had an impact on survival in Hospital 2 (88% versus 79%, respectively, *p* = 0.0409) but not in Hospital 1 (88% versus 86%, respectively, *p* = 0.3469) (**Table 5**). In an attempt to shed further light on the survival differences between Hospital 1 and Hospital 2 according to margin status, patients with margins <5 mm were further stratified using a cut-off of 2 mm (i.e., margins ≤2 mm versus >2* mm*). On analyzing patients with margins <5 mm, we found that Hospital 2 had a higher rate of patients with margins ≤2 mm compared with Hospital 1 (55% versus 34%, respectively, *p <*0.0001; **Table 6**).

**Table 6:** Subgroup analysis of patients treated with surgery alone and margin status <5 mm: additional stratification of margins ≤2 mm versus >2 mm.

Surgery alone /Characteristics	Hospital 1(n = 987)	Hospital 2(n = 553)	*p*
Margin status <5 mm	235	348	<0.0001
≤2 mm	80 (34.0)	191 (54.9)	
>2 mm	155 (66.0)	157 (45.1)	

### Impact of margin status on the treatment modalities implemented in the two hospitals

In the entire cohort, the rate of postoperative adjuvant therapy was found to be lower in patients with margins ≥5 mm than in those with margins <5 mm (42.7% versus 57.0%, respectively, *p <*0.0001). This was more prominent in Hospital 1 (42.9% versus 66.1%, respectively, *p <*0.0001) than in Hospital 2 (42.3% versus 47.6%, respectively, *p* = 0.1032) (**Table 7**).

**Table 7:** Analysis of margin status in relation to treatment modalities implemented in Hospital 1 and Hospital 2.

Characteristic (n, %)	Margin ≥5 mm	Margin <5 mm	*p*
	n (%)	n (%)	
**Hospitals 1 + 2** (n = 3028)			<0.0001
Surgery (1540, 50.9)	957 (57.3)	583 (43.0)	
Surgery plus adjuvant therapy (1488, 49.1)	714 (42.7)	774 (57.0)	
**Hospital 1** (n = 2009)			<0.0001
Surgery (987, 49.1)	752 (57.1)	235 (33.9)	
Surgery plus adjuvant therapy (1022, 50.9)	564 (42.9)	458 (66.1)	
**Hospital 2** (n = 1019)			0.1032
Surgery (553, 54.3)	205 (57.7)	348 (52.4)	
Surgery plus adjuvant therapy (466, 45.7)	150 (42.3)	316 (47.6)	

## Discussion

While outcome differences by treatment center based on case volume are expected in head and neck cancer (2), our current findings that significant survival disparities also exist among patients with OCSCC treated at the two high-volume Taiwanese centers (defined in Taiwan as >78 cases treated per year (13) highlight novel, clinically relevant observations. Of the variables identified in this study as independent predictors of survival outcomes in multivariable analysis, some were patient-related and clearly not modifiable (including tumor subsite, sex, age, pathological T status, pathological N status, pathological stage, tumor differentiation, DOI, and ENE), whereas others were at least in part physician-dependent (including margin status and treatment modality). Overall, our findings support the robustness of the quality criteria mandated by the THPA for OCSCC, including the achievement of a clear margin status (≥5 mm). Of note, these factors are mainly surgeon-dependent and not necessarily related to hospital volume.

The use of a large nationwide database (TCRD) to select the two hospitals included in the study was motivated by 1) the existence of marked inter-hospital differences in terms of margins <5 mm, 2) the need for a comprehensive assessment of the prognostic impact of surgical margins in OCSCC, and 3) the necessity to identify a large number of patients with OCSCC treated in both institutions. In the original cohort, patients treated in Hospital 1 had a higher disease burden at baseline and more frequently received adjuvant therapy. There was also a significant difference with respect to nodal yield during neck dissection, a well-known quality control factor. Differences in disease burden, frequency of adjuvant therapy, and lymph node yield are major contributor to heterogeneous clinical outcomes. While this potential confounder was abrogated by PS matching, the survival differences between patients operated in Hospital 1 and Hospital 2 were even more pronounced in the PS-matched cohort (**Figures 4C, D**). The NCCN guidelines recommend an interval of less than 6 weeks (42 days) between surgery and postoperative RT to enhance locoregional control (6). Compared with Hospital 1, the surgery-to-RT interval was longer in Hospital 2 (both before and after propensity score matching), which might have resulted in a more favorable survival figures in Hospital 1. However, after PS matching, we found no survival differences in patients who received adjuvant RT stratified according to the surgery-to-RT interval (≤42 days versus >42 days; **Figures 3A, B**). Therefore, the surgery-to-RT interval should not be considered as a significant confounding factor affecting the survival outcomes observed in the two hospitals.

The proportion of patients with margins <5 mm was markedly lower in Hospital 1 than in Hospital 2 (34.5% versus 65.2%, respectively) and this was clearly associated with better survival outcomes in the former. These results were confirmed after adjusting for potential confounders in multivariable analysis and by analyzing the PS-matched cohort. The presence of pathological margins <5 mm (including positive margins) is currently recognized as an adverse prognostic factor for patients with OCSCC. However, there is still no consensus in the published literature concerning the most suitable surgical margin. While the NCCN guidelines maintain that OCSCC should be resected with a surgical margin of at least 1−1.5 cm (6), most published studies did not mention how wide surgical margins they used during surgery (14–24). Although we do not have direct information on the surgical principles adopted by the two hospitals, we can infer from the study results that Hospital 2 applied a more conservative than Hospital 1. We also wish to note that the NCCN guidelines included the presence of pathological close margins <5 mm as an adverse risk factor for OCSCC very recently. We therefore believe that our study addresses a clinically relevant and yet still controversial issue among surgeons who treat patients with OCSCC, i.e., how radical should tumor excision be to ensure favorable outcomes? The results of our study show that, despite high volumes, there is still room for improving margin status – an achievement which is expected to be associated with a more favorable prognosis. Of note, the evidence for adjuvant therapy of OCSCC in the NCCN guidelines is derived from patients with head and neck cancer (7, 25, 26). The future inclusion within the NCCN guidelines of appropriate references to consider positive or close margins as an adverse prognostic factor in OCSCC will hopefully mitigate the unfavorable impact of a medically-controllable factor on patient survival (27), even within high-volume hospitals.

One strategy that can potentially benefit the survival of patients with OCSCC is to obtain a surgical margin of at least 1 cm – which would allow achieving pathological clear margins ≥5 mm (28). Unfortunately, the percentage of patients with positive and close margins in previous studies (17–24, 29, 30) remained markedly high (varying from 30% to 60%). This may be at least in part attributed to the adoption of a less radical surgical approach in relation to cosmetic and functional concerns. In the current study, Hospital 2 was clearly less aggressive than Hospital 1, and this may offer an explanation for the less favorable survival figures observed for patients treated in the former. Marginal status had a more significant impact on 5-year survival rates in patients with pStage III-IV disease (compared with pStage I-II) and in those treated with surgery plus adjuvant therapy (compared with surgery alone) in both hospitals. The reason whereby the prognostic impact of the margin status was more pronounced in patients with pStage III-IV disease and in those treated with surgery plus adjuvant therapy may reflect a higher biological tumor aggressiveness in these subgroups. Among patients who were treated with surgery alone, the margin status had an impact on survival in Hospital 2 (*p* = 0.0409) but not in Hospital 1 (*p* = 0.3469). Notably, Hospital 2 had a higher rate of patients with margins ≤2 mm compared with Hospital 1 (66% versus 40%, respectively) and the adverse prognostic significance of margins ≤2 mm is well recognized in the published literature (7, 23, 31–33).

We have previously shown that Taiwanese patients with resected OCSCC who had undergone free flap reconstruction more frequently had clear margins and exhibited a significant survival advantage over those who received local flaps (34). Herein, the use of free-flap reconstructions in Hospital 1 was higher than that observed in Hospital 2 (78.8% [1584/2009] versus 20.8% [212/1019], respectively; *p <*0.0001). On analyzing the entire study cohort, the proportion of margins ≥5 mm was significantly higher in patients who received free-flap reconstructions than in those who did not (63.4% [1139/1796] versus 43.2% [532/1232], respectively, *p <*0.0001). Clearly, a larger resection can have an unfavorable impact on postoperative functional outcomes, especially in terms of swallowing and speech. However, in several studies (35–39), reconstructions with free flaps (*versus* other flaps) have been shown to result in better functional results. In our study, patients with margins ≥5 mm were less frequently treated with postoperative adjuvant therapy than those with margins <5 mm. This phenomenon was more prominent in Hospital 1 than in Hospital 2 – due to the more conservative approach of the latter. Collectively, these results indicate that a clear margin status can reduce the need for adjuvant therapy, ultimately improving quality of life.

While surgical margins and the reconstruction methods may be partly impacted by intrinsic tumor factors and patient characteristics, more experienced surgeons are expected to achieve more radical excisions. Therefore, a beneficial effect of surgical quality assurance on survival through an increase in the rate of clear margins should be expected. However, nodal yield, another quality criterion mandated by the THPA for OCSCC, was not an independent predictor of survival outcomes in the current study. While a higher number of dissected lymph nodes reflects high-quality surgical care, its prognostic impact may be altered by adjuvant therapy – which reduces the risk of regional recurrence associated with low nodal yield. Oral cavity cancer is relatively radioresistant and standard dose fractionation is generally sufficient only for eradicating occult disease. When residual gross nodes are present, dose escalation or higher fraction size regimens are necessary. Based on our clinical experience, conventional fractions of 2 Gy result in either no response or even nodal progression. In general, we adjust both target volume and the dose gradient to suspicious nodes by taking into account the quality of nodal dissections. Under certain circumstances, patients with pN0 do not receive prophylactic irradiation to nodal volumes. Therefore, a high number of dissected lymph nodes does not only guide subsequent clinical management but can also remove gross radioresistant lesions that may have a prognostic significance in certain patients. Importantly, the optimal cutoff for an adequate lymph node yield needs to be further validated in multicenter studies.

There are some limitations to our current investigation. This study was not designed to compare the clinical outcomes between low- and high-volume hospitals. No definite conclusion can currently be reached on this issue for patients with OCSCC (3, 4) and further research is necessary. While being information‐rich, registry studies are prone to inherent and unavoidable confounding factors. In addition, the TCRD had no independent data on local, neck, and distant recurrences; therefore, these endpoints could not be included in the outcome analysis. Due to the inherent limitations of the data sets, we were also unable to analyze the prognostic significance of perineural and lympho-vascular invasion. Finally, unavailability of data in areas where betel quid chewing is uncommon does not permit to generalize our current findings to Western countries. However, our report also has some strengths. This is, to our knowledge, the largest study to date focusing on the prognostic impact of margin status in patients staged according to the AJCC Staging Manual, eight editions; in addition, all participants received a homogeneous approach for primary tumor treatment (lesion excision coupled with neck dissection). On analyzing clinical outcomes, we were also able to adjust for a large number of potential confounders – including tumor subsite, differentiation, DOI, ENE, neck nodal yield, and RT.

## Conclusions

In conclusion, our study shows that, within the two highest-volume hospitals in Taiwan, patients with OCSCC who achieved a clear margin status (≥5 mm) demonstrated more favorable survival outcomes. These results have clinical implications and show how initiatives aimed at improving the margin quality can translate in better outcomes in this clinical population. Importantly, we found that a clear margin status was associated with a reduced use of adjuvant therapy, a factor likely resulting in an improved quality of life. A top-down approach towards the development of policies (i.e., surgical and pathological review of all cases with margins <5 mm), procedures (i.e., implementation of free flap reconstructions), or guidelines (i.e., strict enforcement of surgical margins of at least 1 cm) for appropriate margin standards might be an effective strategy to improve the quality of care in the next future.

## Data availability statement

The original contributions presented in the study are included in the article/Supplementary Material. Further inquiries can be directed to the corresponding author.

## Ethics statement

The studies involving human participants were reviewed and approved by the Institutional Review Board of the Chung Gung Memorial Hospital (reference number: 201801398B0A3) and received a waiver of patient consent. 

## Author contributions

Conception and design: C-TL, L-YL, S-RL AND Y-WW. Analysis and interpretation of data: All authors. Drafting the article or revising it critically for important intellectual content: All authors. Agreement to be accountable for all aspects of the work: All authors. All authors contributed to the article and approved the submitted version.

## Funding

This work was funded by the Chang Gung Medical Research Program (grants CMRPD1H0521 and BMRPC55).

## Acknowledgments

We gratefully acknowledge the Research Service Center for Health Information (Chang Gung University, Taiwan) for assistance with the study design, data management, and statistical analysis.

## Conflict of interest

The authors declare that the research was conducted in the absence of any commercial or financial relationships that could be construed as a potential conflict of interest.

## Publisher’s note

All claims expressed in this article are solely those of the authors and do not necessarily represent those of their affiliated organizations, or those of the publisher, the editors and the reviewers. Any product that may be evaluated in this article, or claim that may be made by its manufacturer, is not guaranteed or endorsed by the publisher.
